# Effects of Bedaquiline on Antimicrobial Activity and Cytokine Secretion of Macrophages Infected with Multidrug-Resistant *Mycobacterium tuberculosis* Strains

**DOI:** 10.1155/2022/2703635

**Published:** 2022-04-11

**Authors:** Xia-Li Lyu, Ting-Ting Lin, Jing-Tao Gao, Hong-Yan Jia, Chuan-Zhi Zhu, Zi-Hui Li, Jing Dong, Qi Sun, Wei Shu, Sai-Sai Wang, Li-Ping Pan, Hai-Rong Huang, Zong-De Zhang, Qi Li

**Affiliations:** Beijing Chest Hospital, Capital Medical University, Beijing Tuberculosis and Thoracic Tumor Research Institute, Beijing 101149, China

## Abstract

**Background:**

Bedaquiline (Bdq) exerts bactericidal effects against drug-susceptible and drug-resistant *Mycobacterium tuberculosis* strains, including multidrug-resistant *M. tuberculosis* strains (MDR-MTBs). However, few reported investigations exist regarding Bdq effects on MDR-MTBs-infected macrophages activities and cytokine secretion. Here, Bdq bactericidal activities against MDR-MTBs and related cellular immune mechanisms were explored.

**Methods:**

Macrophages infected with MDR-MTBs or H37Rv received Bdq treatments (4 h/8 h/24 h/48 h) at 1 × the minimum inhibitory concentration (1 × MIC), 10 × MIC and 20 × MIC. Intracellular colony-forming units (CFUs) and culture supernatant IL-12/23 p40, TNF-*α*, IL-6, and IL-10 were determined using the Luminex® 200^TM^ system. Normally distributed continuous data (mean ± standard deviation) were analyzed using *t-*test or *F*-test (SPSS 25.0, *P* < 0.05 deemed statistically significant).

**Results:**

(1) 100% of Bdq-treated macrophages (all doses applied over 4–48 h) survived with 0% inhibition of proliferation observed. (2) Intracellular CFUs of Bdq-treated MDR-MTBs-infected macrophages decreased over 4–48 h of treatment, were lower than preadministration and control CFUs, decreased with increasing Bdq dose, and resembled H37Rv-infected group CFUs (48 h). (3) For MDR-MTBs-infected macrophages (various Bdq doses), IL-12/23 p40 levels resembled preadministration group levels and exceeded controls (4 h); TNF-*α* levels exceeded preadministration group levels (24 h/48 h) and controls (24 h); IL-12/23 p40 and TNF-*α* levels resembled H37Rv-infected group levels (4 h/8 h/24 h/48 h); IL-6 levels exceeded preadministration and H37Rv-infected group levels (24 h/48 h) and controls (24 h); IL-10 levels resembled preadministration and H37Rv-infected group levels (4 h/8 h/24 h/48 h) and were lower than controls (24 h/48 h); IL-12/23 p40 and IL-10 levels remained unchanged as intracellular CFUs changed, with IL-12/23 p40 levels exceeding controls (4 h) and IL-10 levels remaining lower than controls (24 h/48 h); TNF-*α* and IL-6 levels increased as intracellular CFUs decreased (24 h/48 h) and exceed controls (24 h).

**Conclusion:**

Bdq was strongly bactericidal against intracellular MDR-MTBs and H37Rv in a time-dependent, concentration-dependent manner. Bdq potentially exerted immunomodulatory effects by inducing high-level Th1 cytokine expression (IL-12/23 p40, TNF-*α*) and low-level Th2 cytokine expression (IL-10).

## 1. Introduction

Tuberculosis (TB) is a chronic infectious disease that poses serious treatment challenges, especially for patients with multidrug-resistant *tuberculosis* (MDR-TB) and rifampicin-resistant *tuberculosis* (RR-TB). In fact, the MDR/RR-TB patient treatment success rate is only 57% around the world and 52% in China, as reported in a World Health Organization (WHO) report for 2020 [1]. Therefore, development of new anti-TB drugs is an important strategy for achieving TB control, especially for drug-resistant TB cases. Importantly, during the drug development process, use of biological systems to simulate host–pathogen interactions can reveal drug resistance mechanisms associated with multidrug-resistant *Mycobacterium tuberculosis* (MDR-MTB) strains. Moreover, the use of biologically based disease models enables researchers to determine drug efficacies and mechanisms of action of new anti-TB drugs, so as to provide more effective treatments for achieving MDR-TB control [2].

Bedaquiline (Bdq, TMC207), an ATP synthase inhibitor [3], is effective against drug-susceptible (DS) and drug-resistant *M. tuberculosis* (including MDR-MTB) strains, with several previously reported studies demonstrating clinical efficacy of Bdq and Bdq-containing chemotherapy regimens [4, 5]. In fact, Bdq-containing chemotherapy regimens have entered into the phase III clinical trials according to a 2020 WHO report [1]. However, few reports exist of investigations focused on Bdq effects and mechanism(s) of action when the drug is used to treat macrophages infected with MDR-MTB strains [2]. Therefore, this study aimed to investigate Bdq-associated bactericidal activity and effects on secretion of cytokines by MDR-MTBs-infected macrophages by focusing on certain cytokines with known roles in macrophages activation, phagocytosis, bactericidal activity, and other functions. More specifically, levels of four infrequently reported Th1/Th2 cytokines, IL-12/23 p40, TNF-*α*, IL-6, and IL-10 were measured to assess changes in macrophages cytokine secretion after addition of Bdq to cultures of MDR-MTBs-infected macrophages. The results of this study reveal possible drug-associated cellular immune mechanisms to guide optimization of Bdq administration protocols to maximize drug efficacy.

## 2. Materials and Methods

### 2.1. Compounds, THP-1 Cells, and Bacteria

Bedaquiline (TMC207, Bdq, S5623) was purchased from Selleck (Houston, TX, United States). Alamar Blue was purchased from BD company (Franklin Lakes, New Jersey, United States). The Human Magnetic Luminex® Assay kit was purchased from R&D. Bdq was dissolved in Dimethyl Sulfoxide (DMSO) to generate concentrated stock solutions that were stored without freeze-thaw cycles at −80°C until use. THP-1 cells (an immortalized human monocytic cell line), *M. tuberculosis* H37Rv (ATCC_27294) and MDR-MTB strains (Clinical number 24635) were obtained from Beijing Chest Hospital, Capital Medical University. All MDR-MTB strains were confirmed to be *M. tuberculosis* using para-nitrobenzoic (PNB) testing. Drug resistance to isoniazid and rifampicin was assessed via drug sensitivity testing based on the proportional method, with additional characterization of *rpoB* and *katG* gene mutations conducted via full gene sequencing.

### 2.2. THP-1 Cell Culture, Differentiation, and Bacterial Infection

THP-1 cells were maintained in RPMI-1640 supplemented with 10% FBS at 37°C in a humidified incubator with 5%CO_2_. THP-1 cells were induced to differentiate into macrophages by incubation of cells with 100 ng/ml phorbol 12-myristate 13-acetate (PMA) for 36 h – 48 h prior to their use in experiments; successful differentiation was confirmed based on increased cell adherence (about 70–80%), as assessed via optical microscopy. To generate MTB cultures, bacteria were inoculated into 7H9 medium (containing 10% OADC and 0.05% Tween-80) and then cultures were cultured at 37°C until cells were in logarithmic growth phase (OD_600_ ≈ 0.6–1.0). Thereafter, an ultrasonic disperser was used to disperse and count MTB bacilli. Next, differentiated macrophages were infected with MTB at a multiplicity of infection (MOI) of 10 for 4 h in BSL2 facilities within the Department of Bacteriology of the Beijing Tuberculosis and Thoracic Tumor Research Institute after approval by the Institutional Biosafety Committee of China (ITEM: LA2-6A1 Class II BSC per NSF 49, ULPA filter, ISOCIDE, Asset number: 100408).

### 2.3. Broth Microdilution Minimum Inhibitory Concentration (MIC) Method

A microplate Alamar Blue assay (MABA) was used to determine the minimum inhibitory concentration (MIC) of Bdq for the H37Rv strains. Briefly, the turbidity of the resulting MTB suspension was adjusted with sterile deionized water until it was equivalent to the turbidity of a 1.0 McFarland standard (∼5 × 10^7^ CFU/ml). Next, the MTB suspension was diluted 1 : 50 (∼1 × 10^6^ CFU/ml) in 7H9 broth medium to generate a 2 × stock of the inoculum, which was then transferred to a disposable inoculum reservoir. Next, a volume of 100 µl of the stock inoculum was transferred from the reservoir into each well of a microtiter plate using an eight-channel micropipette (and sterile tips with filters) to deliver a final number of viable bacilli per well of ∼5 × 10^5^ CFU/ml. Control wells for assessing growth of MTB without added Bdq contained macrophages inoculated with MTB, while negative control wells contained macrophages without MTB. Next, Bdq was diluted and tested for activity against MTB based on Clinical and Laboratory Standards Institute (CLSI) guidelines. After the plates were incubated for 7 days at 37°C, 32.5 *μ*l of staining agent (20 *μ*l Alamar Blue and 12.5 *μ*l of 20% Tween-80) was added per well then plates were incubated for another 24 h. Thereafter, wells of the microtiter plates were read by visual inspection to interpret the Bdq MIC results, with MIC defined as the lowest concentration of drug that prevented the color from changing from blue to pink. The MIC of Bdq was 0.03 mg/L.

### 2.4. Measurement of MTB Infection by Colony-forming Units (CFUs) Assays

To assess Bdq effects on MTB survival in macrophages, THP-1 cells (that had been previously induced to differentiate into macrophages) were infected with MTB at a multiplicity of infection of 10 for 4 h, then the cells were washed with phosphate-buffered saline (PBS) four times. Based on a Bdq MIC of 0.03 mg/L, different doses of Bdq (1 × MIC, 10 × MIC, or 20 × MIC) were added to wells and then the plates were incubated for 4 h, 8 h, 24 h, or 48 h. Thereafter, the macrophages were washed again and lysed by addition of PBS containing 0.5% Triton X-100. Next, CFU assays were performed by plating serially diluted suspensions on Middlebrook 7H10 agar plates supplemented with 10% OADC followed by incubation of plates at 37°C for 3 ∼ 4 weeks. After incubation, numbers of MTB colonies representing CFUs within the macrophages were quantified by counting the colonies on the plates.

### 2.5. Magnetic Luminex® Assays

To assess the Bdq effects on MTB-infected macrophages cytokine secretion, differentiated macrophages were infected with MTB at a multiplicity of infection of 10 for 4 h followed by four washes with PBS. Next, various concentrations of Bdq (1 × MIC) were added to wells followed by incubation of plates for 4 h, 24 h, or 48 h. Thereafter, culture supernatants were collected and levels of IL-12/23 p40, TNF-*α*, IL-6, and IL-10 in the supernatants were measured using the Luminex® 200 TM system according to the manufacturer's instructions.

### 2.6. Statistical Analysis

The data were analyzed by SPSS 25.0 software and Prism 8.0 software. The continuous data were expressed as mean ± standard deviation $\left(\bar{x}\pm s\right)$ and analyzed by *t-* or *F*-test. *P* < 0.05 was considered statistically significant.

## 3. Results

### 3.1. Assessment of Bdq Toxicity and Effect on Macrophages Proliferation

As shown in Table 1, OD_450_ values of control (untreated, uninfected macrophages) and experimental wells (Bdq-treated uninfected macrophages) exhibited increasing trends with time. However, for a given timepoint after Bdq addition to wells, decreasing OD_450_ values were observed with increasing Bdq concentration. Thus, the survival rate of Bdq-treated macrophages was 100% and the rate of inhibition of proliferation of Bdq-treated macrophages was 0% for different Bdq concentrations and treatment times.

Intracellular CFUs detected in macrophages infected with H37Rv and MDR-MTB strains after Bdq treatment for 4 h to 48 h.

Bdq exerted potent bactericidal activity against *M. tuberculosis* H37Rv and MDR-MTB bacilli within macrophages, with bactericidal activity assessed by measuring CFU reductions after treatment of infected macrophages with various doses of Bdq for 4 h to 48 h, as shown in Table 2. The results revealed that from 4 h to 48 h after Bdq addition to wells, numbers of intracellular CFUs detected in macrophages infected with MDR-MTB or H37Rv strains decreased gradually with time; CFUs reached their lowest values at 48 h, which were lower than corresponding control group CFU values (infected, untreated) (*P* < 0.05). Notably, when Bdq was administered at doses of 10 × MIC and 20 × MIC, numbers of intracellular CFUs detected in macrophages infected with H37Rv and MDR-MTB strains were lower than CFUs associated with Bdq administration at a dose of 1 × MIC (*P* < 0.05). However, intracellular CFU numbers detected in macrophages infected with H37Rv were similar for Bdq doses of 10 × MIC and 20 × MIC (*P* > 0.05), whereas intracellular CFUs of macrophages infected with MDR-MTBs and treated with a Bdq dose of 20 × MIC were lower than corresponding CFUs in macrophages treated with a Bdq dose of 10 × MIC at all treatment timepoints (*P* < 0.05).

**Figure 1:**
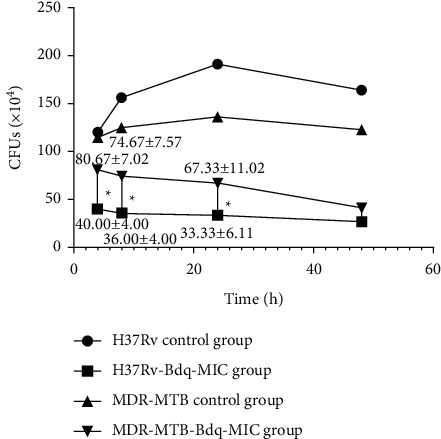
Intracellular CFUs detected in macrophages infected with H37Rv and MDR-MTB strains after Bdq treatment.

Intriguingly, as compared with numbers of intracellular CFUs detected in H37Rv-infected macrophages, numbers of intracellular CFUs detected in MDR-MTBs-infected macrophages were higher at early timepoints after Bdq administration (4 h, 24 h). However, at a later infection stage (48 h) CFUs were similar between the two groups, as shown in Figure 1.

Levels of Th1/Th2 cytokines in culture supernatants of macrophages infected with MDR-MTB strains after Bdq administration.

Tables 3–6 reveal levels of Th1 cytokines (IL-12/23 p40 and TNF*-α*) and Th2 cytokines (IL-6 and IL-10) in culture supernatants of macrophages infected with MDR-MTB and H37Rv strains after Bdq treatment with a 1 × MIC dose (0.03 mg/L) for 4‒48 h.

As shown in Table 3, levels of IL-12/23 p40 in culture supernatants of macrophages infected with MDR-MTB and H37Rv strains did not change before or during Bdq administration over 4–48 h, with no significant difference observed between Bdq-treated macrophages infected with MDR-MTB versus those infected with H37Rv at all timepoints after Bdq administration (*P* > 0.05). However, the level of IL-12/23 p40 in culture supernatants of Bdq-treated macrophages infected with H37Rv was lower than that of the control (untreated) group at 48 h, whereas levels of IL-12/23 p40 in culture supernatants of macrophages infected with MDR-MTBs were higher than that of the control group at 4 h after Bdq administration (*P* < 0.05).

As shown in Table 4, levels of TNF-*α* in culture supernatants of macrophages infected with MDR-MTB and H37Rv strains at 24 h and 48 h were higher than corresponding preadministration group levels, with no significant difference observed between macrophages infected with MDR-MTB and H37Rv at all treatment timepoints (*P* > 0.05). Meanwhile, TNF-*α* levels in culture supernatants of MDR-MTBs-infected macrophages were higher than the corresponding control group level after 24-h Bdq treatment (*P* < 0.05).

As shown in Table 5, IL-6 levels in culture supernatants of macrophages infected with MDR-MTB and H37Rv strains at 24 h and 48 h after Bdq administration were higher than corresponding preadministration group levels. Meanwhile, IL-6 levels in culture supernatants of MDR-MTBs-infected macrophages were higher than corresponding levels in culture supernatants of H37Rv-infected macrophages after 24-h and 48-h Bdq treatment (*P* < 0.05), whereas IL-6 levels in culture supernatants of H37Rv-infected macrophages were lower than corresponding control group levels after 24-h and 48-h Bdq treatment. Notably, IL-6 levels in culture supernatants of MDR-MTBs-infected macrophages were higher than the corresponding control group level after 24-h Bdq treatment (*P* < 0.05).

As shown in Table 6, IL-10 levels in culture supernatants of macrophages infected with MDR-MTB and H37Rv strains remained unchanged before and after Bdq administration for 4–48 h, with no significant differences observed between macrophages infected with MDR-MTBs and H37Rv at any timepoint (*P* > 0.05). In addition, IL-10 levels in culture supernatants of macrophages infected with H37Rv were lower than corresponding control group levels after 48 h Bdq treatment, although IL-10 levels in culture supernatants of MDR-MTB-infected macrophages were lower than those of the control group after 24-h and 48-h Bdq treatments (*P* < 0.05).

Analysis of the relationship between Th1/Th2 cytokine level and intracellular CFUs of MDR-MTBs-infected macrophages after Bdq administration.

**Figure 2:**
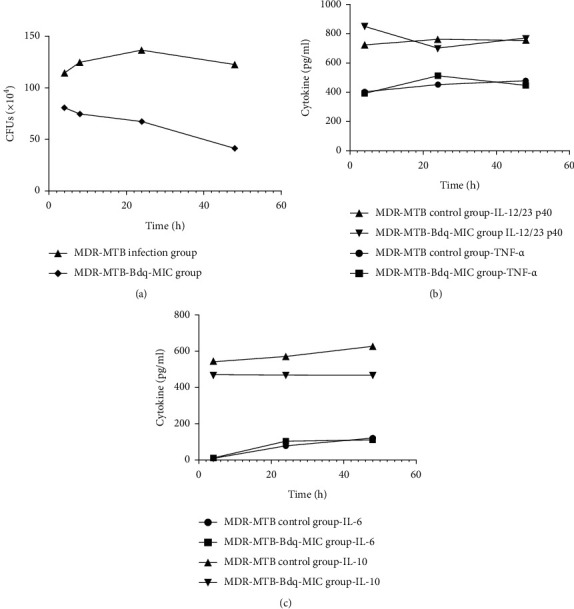
The relationship between Th1/Th2 cytokine levels and intracellular CFUs of MDR-MTBs-infected macrophages after Bdq administration. (a) Intracellular CFUs detected in macrophages infected with MDR-MTB after Bdq treatment. (b) Levels of Th1 cytokine in culture supernatant of macrophages infected with MDR-MTB after Bdq administration. (c) Levels of Th2 cytokine in culture supernatants of macrophages infected with MDR-MTB after Bdq administration.

Results revealing the relationship between Th1/Th2 cytokine level changes and numbers of intracellular CFUs detected in MDR-MTBs-infected macrophages after administration of a Bdq dose of 1 × MIC over 4–48 h are shown in Figure 2. Levels of IL-12/23 p40 and IL-10 remained unchanged as intracellular CFU numbers detected in MDR-MTBs-infected macrophages decreased after Bdq administration. Meanwhile, IL-12/23 p40 levels were greater than the corresponding control group level at 4 h after Bdq administration, whereas IL-10 levels were lower than corresponding control group levels at 24 h and 48 h. Moreover, levels of TNF-*α* secreted by MDR-MTBs-infected macrophages at 24 h and 48 h increased as intracellular CFUs decreased and exceeded the control group TNF-*α* level after 24 h of Bdq treatment. In addition, at all Bdq treatment timepoints, levels of IL-6 secreted by MDR-MTBs-infected macrophages increased as intracellular CFU numbers decreased, as observed for the control group.

## 4. Discussion

*M. tuberculosis* (MTB), a species of facultative bacteria, infects macrophages. Macrophages, which are derived from monocytes, have powerful phagocytic functions that enable them to act as important immune effector cells against MTB invasion. Nevertheless, it has been reported that infection with MDR-MTB strains can lead to abnormal host cellular immune functions that can limit killing and elimination of MDR-MTB bacilli from the host [6, 7], prompting researchers to search for drugs to restore immune function. One such drug, Bdq, holds promise, but few reported studies have investigated Bdq effects on secretion of cytokines by macrophages infected with MDR-MTB strains. Therefore, in this study, we determined whether cytokines secreted by macrophages participate in the regulation of Bdq bactericidal activity.

As shown in Table 1, the survival rate of macrophages after exposure to Bdq was 100% and the Bdq-associated inhibition rate of macrophages proliferation was 0% for different Bdq concentrations and exposure times. Thus, these results suggest that exposure of macrophages to Bdq had no significant effect on macrophages survival and proliferation.

As shown in Table 2, numbers of detected intracellular CFUs of MDR-MTBs-infected macrophages were lower than corresponding control (untreated) group CFU numbers at 8 h, 24 h, and 48 h after Bdq addition to cultures (*P* < 0.05). These results thus suggest that Bdq exerted good bactericidal activity against MDR-MTB within macrophages that exhibited several important characteristics. First, Bdq exerted early bactericidal activity against intracellular bacilli of MDR-MTB strains, as reflected by 30% decreases in numbers of CFUs detected within MDR-MTBs-infected macrophages at 4 h after Bdq administration. This result was consistent with results reported by Diacon et al. [8], which demonstrated that average sputum CFU numbers were reduced by about 25% in 23 MDR-TB patients treated with Bdq for 1 week, thus suggesting that early administration of Bdq could kill MDR-MTB bacilli while also potentially reducing bacterial resistance. Second, the bactericidal activity of Bdq was time-dependent, whereby intracellular CFUs representing intracellular bacilli in MDR-MTBs-infected macrophages decreased by 64% at 48 h after Bdq administration relative to CFU numbers detected during the preadministration stage, thus suggesting that Bdq exhibited a delayed bactericidal effect. This result aligned with results reported by Rustomjee et al. [9], which demonstrated that numbers of CFUs in sputum samples obtained from smear-positive pulmonary TB patients decreased rapidly after treatment with Bdq for 4 days. Third, Bdq bactericidal activity was concentration-dependent (shown in Table 2), whereby intracellular CFUs of MDR-MTBs-infected macrophages were reduced in a Bdq dose-dependent manner with increasing Bdq dose (1 × MIC, 10 × MIC, 20 × MIC). Meanwhile, numbers of intracellular CFUs detected in macrophages administered a 20 × MIC Bdq dose were lower than those of macrophages administered a 10 × MIC dose at all timepoints after Bsq administration. These results were consistent with results reported by Rustomjee et al. [9], which indicated that numbers of CFUs in patient sputa were lower after treatment with a 400-mg/day dosage of Bdq for 7 days as compared to CFU numbers detected in sputa of patients treated with Bdq dosages of 100 mg/day and 25 mg/day. Taken together, these results suggest that use of a reasonable therapeutic dose of Bdq can support effective bactericidal activity. Fourth, Bdq bactericidal activity against MDR-MTB strains was similar to its activity against the H37Rv strain during late-stage (48 h) treatment, as shown in Figure 1, although numbers of intracellular CFUs detected in MDR-MTBs-infected macrophages were higher than corresponding numbers detected in H37Rv-infected macrophages at 4–24 h after Bdq administration. This result aligned with results reported by Tweed et al. [10], which indicated that the sputum conversion rate from positive to negative was similar between DS-TB and MDR-TB patients after treatment with a chemotherapeutic regimen that included a 200-mg/day dosage of Bdq for 56 days [76.1% (95% CI: 64.0 ∼ 88.3) vs. 79.8% (95%CI: 2.4 ∼ 97.2)], thus suggesting that the Bdq treatment course used for MDR-TB patients should be longer than that used for treating DS-TB patients.

Unlike most other pathogenic bacteria, MTB bacilli mainly target and enter pulmonary macrophages that, in turn, react by suppressing MTB activities or by killing intracellular MTB through secretion of cytokines that influence expression and/or activation of host metabolic pathways, autophagy, pyrolysis, and other host activities [11]. In this study, macrophages secretion of four Th1/Th2-related cytokines was monitored to explore Bdq effects on macrophages cytokine secretion and to identify possible synergistic mechanisms involved in macrophage-based killing of MDR-MTB.

In terms of Th1 cytokines, IL-12/23 p40 and TNF-*α* exert proinflammatory effects by promoting production of IFN-*γ* or by coordinating with IFN-*γ*. In turn, IFN-*γ* acts to directly or indirectly promote macrophages activation that can lead to enhanced phagocytosis of MTB, inhibition of anti-MTB activities, and MTB killing [12, 13]. Results shown in Tables 3 and 4 indicate that levels of IL-12/23 p40 and TNF-*α* in culture supernatants of MDR-MTBs-infected macrophages were higher than corresponding control group levels at 4 h and 24 h after Bdq administration. These results thus suggest that Bdq acted as an immunopotentiator by promoting production of IL-12/23 p40 and TNF-*α* to coordinate and enhance the macrophage-induced bactericidal effect. Nevertheless, this Bdq effect was short-lived, a result likely due to the fact that Bdq was administered as a one-time, short-term treatment that could not sustain long-term cytokine secretion. Moreover, additional mechanisms related to the delay in the observed Bdq bactericidal effect may have contributed to the short-term nature of the Bdq effect, warranting further study. Furthermore, levels of IL-12/23 p40 and TNF-*α* after Bdq treatment were similar between culture supernatants of macrophages infected with MDR-MTB and H37Rv strains, suggesting that the Bdq immunomodulatory effect against MTB was not influenced by MTB drug resistance.

In terms of Th2 cytokines, IL-6 and IL-10 are known to inhibit IFN-*γ*-induced macrophages phagosome-lysosome fusion and autophagy, so as to provide favorable conditions for MTB survival in vivo [14, 15]. However, results obtained here (Tables 5 and 6) indicated that IL-10 levels in culture supernatants of MDR-MTBs-infected macrophages were lower than those of the control group after 24 h and 48 h Bdq treatment. Thus, Bdq may have exerted an immunosuppressive effect on IL-10 secretion that ultimately promoted the development of an immune response dominated by Th1 cells that indirectly eliminated MDR-MTB bacilli. Nevertheless, it is possible that decreased bacterial load resulting from Bdq-associated killing of MDR-MTB bacilli within macrophages may have led to reduced stimulation of Th2 cytokine production. In addition, IL-6 levels in culture supernatants of MDR-MTBs-infected macrophages were higher than corresponding levels in culture supernatants of H37Rv-infected macrophages after 24 h and 48 h Bdq treatments, whereas IL-10 levels were similar to those of H37Rv-infected macrophages at all treatment timepoints. Taken together, these results suggest that Bdq effects on macrophages secretion of different cytokines differed depending on whether the macrophages were infected with MDR-MTB or H37Rv strains to explain inconsistencies in secreted IL-6 and IL-10 profiles for different MTB strains, warranting further study.

In addition to assessing secreted cytokine levels, we studied effects of four cytokines on phagocytic and bactericidal functions of MDR-MTBs-infected macrophages, as shown in Figure 2. Levels of IL-12/23 p40 and IL-10 secreted by MDR-MTBs-infected macrophages remained unchanged as intracellular CFUs decreased. Meanwhile, levels of IL-12/23 p40 secreted by MDR-MTBs-infected macrophages after 4 h Bdq treatment were higher than that of the control group, whereas levels of IL-10 after 24 h and 48 h of treatment were lower than corresponding control group levels. Moreover, secreted TNF-*α* levels after 24 h and 48 h Bdq treatments increased as intracellular CFUs decreased, with a higher TNF-*α* level after 24-h treatment observed as compared to that of the control group. Taken together, these results suggest that Bdq may exert a regulatory effect on macrophages cytokine secretion by upregulating secretion of IL-12/23 p40 and TNF-*α* and downregulating secretion of IL-10 to promote a Th1 immune response that could ultimately enhance macrophages phagocytic and bactericidal functions. By contrast, although secreted IL-6 levels of MDR-MTBs-infected macrophages increased as intracellular CFUs decreased, IL-6 levels resembled those of the control group at all Bdq-treatment timepoints, thus suggesting that Bdq had no regulatory effect on IL-6 levels during MDR-MTBs growth and reproduction in macrophages. This result was consistent with results reported by Li et al. [16], Doan et al. [17], and Ban et al. [18] that together suggest that Bdq treatment directly alters macrophages-associated regulation of immune system functions to enhance drug therapeutic effects or reduce host susceptibility to invading pathogens to ultimately achieve a strong bactericidal effect.

This study had several limitations. First, our results only demonstrated Bdq effects for macrophages infected with a limited set of similar MDR-MTB clinical isolates. Thus, we should expand the sample size of clinical isolates and number of drug resistance types in order to obtain more reliable results. Moreover, we studied a limited range of Bdq doses and thus plan to measure cytokine levels after exposure of macrophages to higher Bdq doses in the future. Furthermore, we will consider verifying that Bdq effects on cytokine levels can be replicated in vivo by conducting animal studies in the future.

In conclusion, Bdq exerted a strong bactericidal activity against intracellular MDR-MTB. This Bdq effect was time-dependent and concentration-dependent, with late bactericidal activity observed against MDR-MTB that resembled bactericidal activity against H37Rv. Mechanistically, the Bdq-induced bactericidal effect may have been due to drug-induced immunomodulation that led to increased expression of IL-12/23 p40 and TNF-*α* and reduced expression of IL-10.

## Figures and Tables

**Table 1:** Cytotoxicity test after Bdq treatments (OD_450_, $\bar{x}\pm s$).

	4 h	8 h	24 h	48 h	*F* test	*P* value
Blank well	0.19 ± 0.00	0.20 ± 0.00	0.21 ± 0.00	0.25 ± 0.00	302.383	0.001
Control well	2.61 ± 0.07	2.75 ± 0.01	3.28 ± 0.00	3.63 ± 0.01	582.410	0.001
Experiment well (MIC)	3.00 ± 0.00	3.94 ± 0.04	4.02 ± 0.05	4.21 ± 0.01	770.868	0.001
Experiment well (10 MIC)	2.98 ± 0.01	3.17 ± 0.02	3.70 ± 0.01	4.17 ± 0.02	3496.973	0.001
Experiment well (20 MIC)	2.64 ± 0.01	2.93 ± 0.02	3.67 ± 0.02	3.98 ± 0.04	1965.094	0.001
*F* test	4501.332	11129.366	11417.576	21747.170	—	—
*P* value	0.001	0.001	0.001	0.001	—	—
Survival rate (MIC)	100.00	100.00	100.00	100.00	—	—
Survival rate (10 MIC)	100.00	100.00	100.00	100.00	—	—
Survival rate (20 MIC)	100.00	100.00	100.00	100.00	—	—
Inhibition rate (MIC)	0	0	0	0	—	—
Inhibition rate (10 MIC)	0	0	0	0	—	—
Inhibition rate (20 MIC)	0	0	0	0	—	—

Survival rate = [(experiment well-blank well)/(control well-blank well)] × 100%. Inhibition rate = [(control well-experiment well)/(control well-blank well)] × 100%.

**Table 2:** Intracellular CFUs detected in macrophages infected with H37Rv and MDR-MTB strains after Bdq treatment $\left(\bar{x}\pm s\right)$.

	4 h before administration	4 h after administration	8 h after administration	24 h after administration	48 h after administration	*F* test	*P* value
H37Rv strains							
Control	120.00 ± 4.00	104.67 ± 9.87	114.00 ± 5.29	102.67 ± 13.61^*#*^	85.33 ± 6.43^#&♣^	7.111	0.006
MIC	120.00 ± 4.00	40.00 ± 4.00^*a*#^	36.00 ± 4.00^*a*#^	33.33 ± 6.11^*a*#^	26.67 ± 11.55^*a*#&^	103.085	0.001
10 MIC	120.00 ± 4.00	33.33 ± 6.11^*a*#^	22.67 ± 4.62^*ab*#&^	17.33 ± 2.31^*ab*#&^	14.67 ± 4.16^*ab*#&^	303.890	0.001
20 MIC	120.00 ± 4.00	26.67 ± 6.11^*ab*#^	24.00 ± 6.93^*ab*#^	21.33 ± 2.31^*ab*#^	10.67 ± 1.16^*ab*#&♣*∗*^	279.210	0.001
*F*-test	—	83.090	201.612	81.943	74.621	—	—
*P* value	—	0.001	0.001	0.001	0.001	—	—
MDR-MTB strains							
Control	114.67 ± 7.57	92.67 ± 6.43^*#*^	102.67 ± 4.62	96.67 ± 11.55^*#*^	83.33 ± 5.77^#♣^	7.128	0.006
MIC	114.67 ± 7.57	80.67 ± 7.02^*#*^	74.67 ± 7.57^*a*#^	67.33 ± 11.02^*a*#^	41.33 ± 2.31^*a*#&♣*∗*^	36.062	0.001
10 MIC	114.67 ± 7.57	74.67 ± 7.57^*a*#^	53.33 ± 3.06^*ab*#&^	34.67 ± 4.62^*ab*#&♣^	30.00 ± 7.21^*ab*#&♣^	90.818	0.001
20 MIC	114.67 ± 7.57	42.00 ± 13.12^*abc*#^	28.00 ± 6.93^*abc*#&^	22.00 ± 2.00^*abc*#&^	19.33 ± 1.16^*abc*#&^	84.097	0.001
*F* test	—	17.604	88.732	48.437	102.507	—	—
*P* value	—	0.001	0.001	0.001	0.001	—	—

Control: No drug on macrophages infected by MTB. ^*#*^: The *P* value of 4 h before administration and other group was less than 0.05. ^&^: The *P* value of 4 h after administration and other group was less than 0.05. ^♣^: The *P* value of 8 h after administration and other group was less than 0.05. ^*∗*^: The *P* value of 24 h after administration and other group was less than 0.05. ^*a*^: The *P* value of control and other group was less than 0.05. ^*b*^: The *P* value of MIC and other group was less than 0.05. ^*c*^: The *P* value of 10MIC and other group was less than 0.05.

**Table 3:** Levels of IL-12/23 p40 in culture supernatants of macrophages infected with H37Rv and MDR-MTB strains after Bdq administration (pg/ml, $\bar{x}\pm s$).

	4 h before administration	4 h after administration	24 h after administration	48 h after administration	*F* test	*P* value
H37Rv strains						
Control	737.87 ± 93.61	722.80 ± 2.70	875.86 ± 21.11	867.81 ± 1.63	5.834	0.061
Bdq	737.87 ± 93.61	749.83 ± 55.78	795.97 ± 16.55	797.54 ± 5.12	0.629	0.633
*t*-test	—	−0.684	4.211	18.491	—	—
*P* value	—	0.564	0.052	0.003	—	—
MDR-MTB strains						
Control	757.70 ± 52.47	722.54 ± 2.64	760.78 ± 0.61	752.17 ± 1.80	0.890	0.519
Bdq	757.70 ± 52.47	852.42 ± 1.85	699.80 ± 61.95	767.13 ± 70.27	2.746	0.177
*t*-test	—	−56.980	1.392	−0.301	—	—
*P* value	—	0.001	0.299	0.792	—	—

**Table 4:** Levels of TNF-*α* in culture supernatants of macrophages infected with H37Rv and MDR-MTB strains after Bdq administration (pg/ml, $\bar{x}\pm s$).

	4 h before administration	4 h after administration	24 h after administration	48 h after administration	*F* test	*P* value
H37Rv strains						
Control	307.95 ± 17.27	385.20 ± 1.52^*#*^	500.39 ± 0.37^#&^	436.28 ± 0.57^#&♣^	175.914	0.001
Bdq	307.95 ± 17.27	382.50 ± 9.66	407.93 ± 53.18^*#*^	444.71 ± 8.41^#^	8.129	0.035
*t*-test	—	0.390	2.459	−1.415	—	—
*P* value	—	0.734	0.133	0.293	—	—
MDR-MTB strains						
Control	334.96 ± 9.33	402.47 ± 2.85^*#*^	451.83 ± 1.19^#&^	475.95 ± 0.54^#&♣^	320.338	0.001
Bdq	334.96 ± 9.33	392.36 ± 45.41	514.09 ± 10.16^#&^	447.54 ± 11.60^*#*^	19.648	0.007
*t*-test	—	0.314	−8.606	3.461	—	—
*P* value	—	0.783	0.013	0.074	—	—

**Table 5:** Levels of IL-6 in culture supernatants of macrophages infected with H37Rv and MDR-MTB strains after Bdq administration (pg/ml, $\bar{x}\pm s$).

	4 h before administration	4 h after administration	24 h after administration	48 h after administration	*F* test	*P* value
H37Rv strains						
Control	9.75 ± 2.91	11.05 ± 1.07	59.96 ± 0.35^#&^	87.06 ± 0.37^#&♣^	1171.658	0.001
Bdq	9.75 ± 2.91	11.81 ± 0.00	46.36 ± 0.00^#&^	61.95 ± 3.17^#&♣^	288.665	0.001
*t*-test	—	−1.000	54.400	11.135	—	—
*P* value	—	0.423	0.001	0.008	—	—
MDR-MTB strains						
Control	7.69 ± 0.00	13.45 ± 0.64^*#*^	77.87 ± 0.21^#&^	119.50 ± 0.84^#&♣^	19757.538	0.001
Bdq	7.69 ± 0.00	11.81 ± 0.00	105.09 ± 0.00^#&^	107.40 ± 9.75^#&^	261.443	0.001
*t*-test	—	3.593	−181.467	1.748	—	—
*P* value	—	0.069	0.001	0.223	—	—

**Table 6:** Levels of IL-10 in culture supernatants of macrophages infected with H37Rv and MDR-MTB strains after Bdq administration (pg/ml, $\bar{x}\pm s$).

	4 h before administration	4 h after administration	24 h after administration	48 h after administration	*F* test	*P* value
H37Rv strains						
Control	465.43 ± 5.95	528.99 ± 1.70^*#*^	582.81 ± 3.58^#&^	566.52 ± 0.25^#&♣^	425.322	0.001
Bdq	465.43 ± 5.95	483.38 ± 43.61	431.66 ± 87.93	440.30 ± 25.79	0.432	0.742
*t*-test	—	1.478	2.429	6.921	—	—
*P* value	—	0.277	0.136	0.020	—	—
MDR-MTB strains						
Control	525.71 ± 79.17	544.22 ± 0.80	571.63 ± 1.93	627.78 ± 4.40	2.517	0.197
Bdq	525.71 ± 79.17	470.29 ± 32.51	471.07 ± 15.67	466.71 ± 16.06	0.815	0.549
*t*-test	—	3.215	9.005	13.680	—	—
*P* value	—	0.085	0.012	0.005	—	—

## Data Availability

The data can be obtained from the corresponding author upon reasonable request.

## Abbreviations

MTB: *Mycobacterium tuberculosis*
MDR-MTB: Multidrug-resistant *Mycobacterium tuberculosis*
MDR-TB: Multidrug-resistant *tuberculosis*
RR-TB: Rifampicin-resistant *tuberculosis*
Bdq: Bedaquiline
CFUs: Colony-forming units
MIC: Minimum inhibitory concentration.
